# Asymmetric reproductive barriers and mosaic reproductive isolation: insights from Misty lake–stream stickleback

**DOI:** 10.1002/ece3.1012

**Published:** 2014-03-10

**Authors:** Katja Räsänen, Andrew P Hendry

**Affiliations:** Redpath Museum and Department of Biology, McGill University859 Sherbrooke St. W, Montréal, QC, H3A 2K6, Canada

**Keywords:** Adaptive divergence, ecological speciation, fish, reproductive isolation

## Abstract

Ecological speciation seems to occur readily but is clearly not ubiquitous – and the relative contributions of different reproductive barriers remain unclear in most systems. We here investigate the potential importance of selection against migrants in lake/stream stickleback (*Gasterosteus aculeatus*) from the Misty Lake system, Canada. This system is of particular interest because one population contrast (Lake vs. Outlet stream) shows very low genetic and morphological divergence, whereas another population contrast (Lake vs. Inlet stream) shows dramatic genetic and morphological divergence apparently without strong and symmetric reproductive barriers. To test whether selection against migrants might solve this “conundrum of missing reproductive isolation”, we performed a fully factorial reciprocal transplant experiment using 225 individually marked stickleback collected from the wild. Relative fitness of the different ecotypes (Lake, Inlet, and Outlet) was assessed based on survival and mass change in experimental enclosures. We found that Inlet fish performed poorly in the lake (selection against migrants in that direction), whereas Lake fish outperformed Inlet fish in all environments (no selection against migrants in the opposite direction). As predicted from their phenotypic and genetic similarity, Outlet and Lake fish performed similarly in all environments. These results suggest that selection against migrants is asymmetric and, together with previous work, indicates that multiple reproductive barriers contribute to reproductive isolation. Similar mosaic patterns of reproductive isolation are likely in other natural systems.

## Introduction

Ecological speciation occurs when reproductive isolation arises due to adaptive divergence between populations inhabiting ecologically different environments (Schluter 2000; Nosil 2012). This process now has considerable empirical support from a wide range of taxa (Schluter 2000; Rundle and Nosil 2005; Funk et al. 2006; Nosil 2012; Shafer and Wolf 2013) – yet it is clearly not ubiquitous. For instance, a growing number of studies seeking evidence of reproductive barriers between populations in different environments have failed to find them or have found that they are very weak (reviews: Hendry 2009; Nosil et al. 2009). At the same time, a growing number of studies report speciation in the apparent absence of ecological differences (Rundell and Price 2009; Svensson 2012). These variable results highlight the value of considering the relative contributions of multiple reproductive barriers in taxa that vary in their progress toward speciation – whether ecological or otherwise. Such analyses should prove useful in attempting to delineate the conditions that do and do not promote ecological speciation – and the combinations of reproductive barriers that are most important.

A reproductive barrier that should be particularly powerful and ubiquitous is selection against migrants, which occurs when individuals adapted to one environment (or habitat or resource) immigrate to (or start to use) another environment (Hendry 2004; Nosil et al. 2005). In such cases, local adaptation is expected to reduce the fitness of immigrants through either lower survival (“immigrant inviability”: Nosil et al. 2005) or lower fecundity or mating success (“immigrant infecundity”: Smith and Benkman 2007). Selection against migrants is expected to be important and common for several reasons. First, it often acts early in the life cycle (e.g., cross-type mating would usually occur later) and so is expected to capture more of the total isolation (Nosil et al. 2005). Second, it acts as an “automatic magic trait” in that divergent selection acts directly on the traits that also influence reproductive isolation (Servedio et al. 2011). Third, the many reciprocal transplant experiments that have been conducted across diverse organisms frequently find lower survival or fecundity in individuals moved between environments (Schluter 2000; Nosil et al. 2005; Hereford 2009).

**Figure 1:**
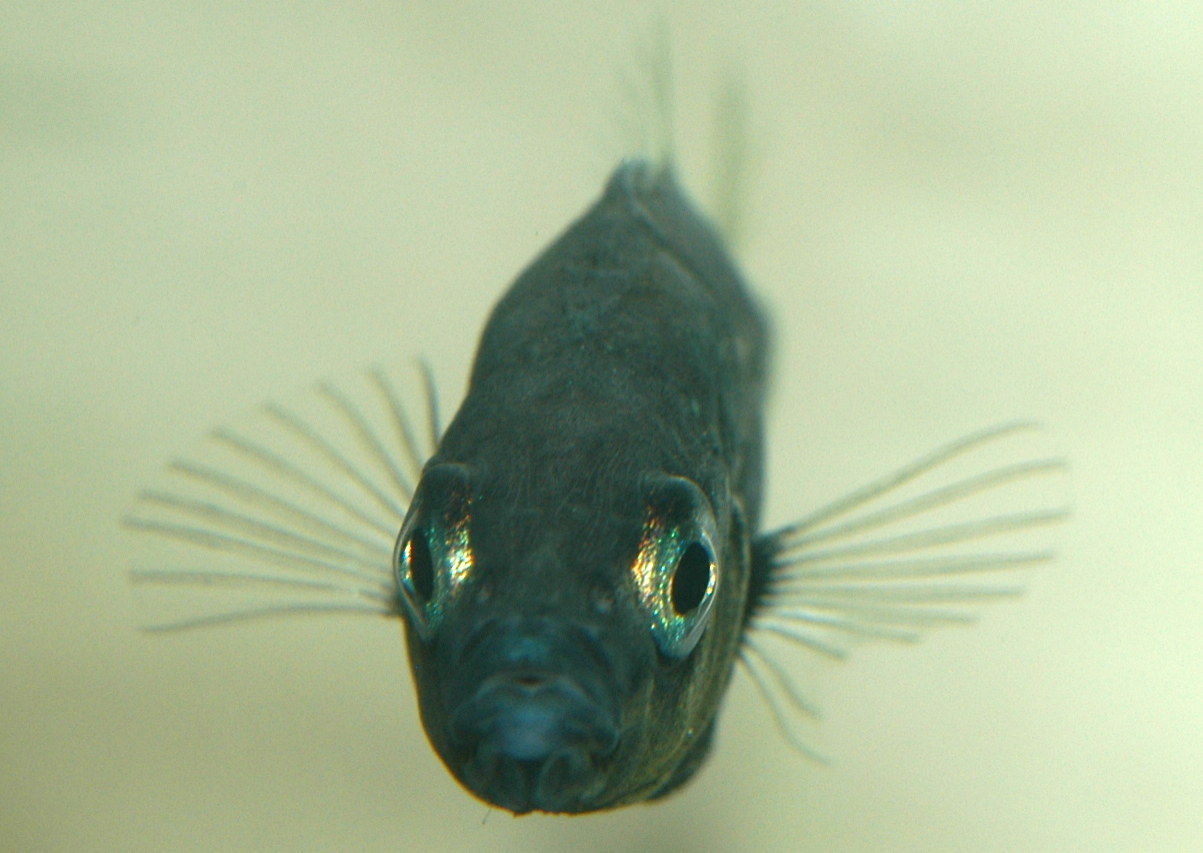
A male threespine stickleback from the Misty system. Copyright: A. P. Hendry.

Two contrasting predictions can be made in the context of selection against migrants. First, populations living in very different environments and showing strong divergence in adaptive traits should show local superiority (e.g., survival and growth should be higher for local than for immigrant individuals in a given environment; Kawecki and Ebert 2004; Nosil et al. 2005; Hereford 2009; Blanquart et al. 2013). Second, populations showing low divergence in adaptive traits should show small (if any) differences in fitness between local and immigrant individuals in a given environment. The second situation might occur if the environments are not very divergent or if adaptive divergence is constrained for some reason, such as high gene flow (Räsänen and Hendry 2008). The latter context is particularly interesting, because it presents a case where ecological speciation might be predicted (divergent selection is strong) but cannot be achieved. We test these two predictions in lake/stream threespine stickleback (*Gasterosteus aculeatus;* Fig. 1) from the Misty Lake watershed in British Columbia, Canada.

### Lake/stream stickleback

Threespine stickleback are a good model for studying progress toward ecological speciation because they show dramatic adaptive divergence between populations in different environments (review: Bell and Foster 1994; McKinnon and Rundle 2002), but highly variable progress toward ecological speciation (Berner et al. 2009; Hendry et al. 2009). That is, in some population contrasts, reproductive barriers can be very strong, whereas in others, similar barriers can be weak or absent (e.g., Jones et al. 2008; Hendry et al. 2009; Raeymaekers et al. 2010; Räsänen et al. 2012). This variation provides excellent opportunities to uncover the factors that promote and constrain progress toward ecological speciation (Hendry et al. 2009).

Our investigation focuses on parapatric stickleback populations in lake versus stream environments. Suitable properties of this system include (1) independent evolutionary origins of lake/stream pairs in many different watersheds (Hendry and Taylor 2004; Berner et al. 2009), (2) strongly divergent foraging environments that generate strong divergent selection (Lavin and McPhail 1993; Berner et al. 2008; Kaeuffer et al. 2012), (3) high gene flow that can sometimes constrain lake/stream divergence (Hendry et al. 2002; Hendry and Taylor 2004; Moore et al. 2007), and (4) highly variable progress toward ecological speciation (Berner et al. 2009, 2010; Roesti et al. 2012). Ecological reproductive barriers are likely: habitat preferences can be important (Bolnick et al. 2009), reproductive timing differences are likely (A. Hendry & D. Bolnick, pers. obs.), and Lake fish have difficulty swimming upstream (Hendry et al. 2002). In contrast, strong genetic incompatibilities are unlikely, given that among ecotype crosses can be successfully conducted and hybrids from these crosses are viable in the laboratory (Lavin and McPhail 1993; Raeymaekers et al. 2010; Berner et al. 2011). Overall, many of these reproductive barriers appear rather weak in at least some places, and assortative mating has not been found (Raeymaekers et al. 2010; Räsänen et al. 2012).

The present study was conducted in the Misty watershed, where two stream populations (Inlet and Outlet) are found in parapatry with the Lake population. The Inlet and Lake populations show very low gene flow (as inferred from neutral markers) and strong genetically based adaptive divergence in a broad suite of phenotypic traits (Hendry et al. 2002; Delcourt et al. 2008; Sharpe et al. 2008; Raeymaekers et al. 2009; Berner et al. 2011; Hendry et al. 2011; Kaeuffer et al. 2012; Baker et al. 2013). Despite this genetic and phenotypic divergence, strong symmetric reproductive barriers have not yet been found in the Misty system, leading Räsänen et al. (2012) to pose the “conundrum of missing reproductive isolation.” The Outlet and Lake populations also experience divergent selection but, in contrast to the Inlet and Lake populations, do not show much adaptive divergence owing to very high gene flow (Hendry et al. 2002; Moore et al. 2007; Delcourt et al. 2008; Sharpe et al. 2008; Berner et al. 2008, 2009; Roesti et al. 2012).

We here use a reciprocal transplant experiment in the wild with individually marked fish placed in enclosures to test for selection against migrants between these two Misty lake/stream population pairs. First, we ask whether trait differences predict performance differences: that is, Lake and Inlet fish should perform differently (measured as survival and mass change), whereas Lake and Outlet fish should perform similarly. Second, we ask whether selection against migrants is evident: that is, Lake fish should perform better than Inlet fish in the lake whereas Inlet fish should perform better than Lake fish in the inlet (no such differences should be evident in Lake/Outlet contrasts). Importantly, our study used wild-caught fish. Although this means that any performance differences cannot be conclusively ascribed to genetic differences, genetic differences do seem likely given the documented genetic basis for adaptive trait divergence in this system (Lavin and McPhail 1993; Hendry et al. 2002; Delcourt et al. 2008; Raeymaekers et al. 2009, 2010; Berner et al. 2011; Hendry et al. 2011; Baker et al. 2013). Moreover, the use of wild-caught fish in reciprocal transplant experiments is usually the starting point for such studies (e.g., 66.7% of the studies in the meta-analysis of Hereford 2009). Finally, and most importantly, the use of wild-caught fish (as opposed to common garden fish) encompasses the effects of the whole phenotype and, hence, is most directly relevant to selection against migrants in nature.

## Material and Methods

### The experiment

The experiment consisted of a fully reciprocal transplant experiment conducted in enclosures in the wild. In each of the three environments (lake, outlet, and inlet), experimental sites were chosen based on prior knowledge of appropriate stickleback habitat and their suitability for enclosures (water depths of 40–60 cm and low flow rates). In each of the environments, a single large enclosure (approximate sizes dictated by available space: inlet: 9.9 m^2^; lake: 13.5 m^2^; outlet: 7.8 m^2^) consisting of white nylon mesh (Delta, 10 mm diameter; Nylonnet Co., Memphis, TN) was erected. These enclosures and their construction were similar to – although larger and with more stickleback – than those in several previous studies (e.g., Hatfield and Schluter 1999; Rundle 2002; Hendry et al. 2002). Ideally, we would have used multiple (replicate) enclosures per environmental type. However, as enclosure number trades-off with enclosure size, we elected to use a single large enclosure per habitat (as opposed to many small as used by Hendry et al. 2002), to provide an environment that would more closely mimic a natural migration event (i.e., bracketing the local habitat heterogeneity, and allowing for habitat choice and competitive interactions within groups of stickleback).

The bottom edge of the mesh of the enclosures was buried in gravel, and the upper end was suspended well above the surface with posts hammered into the substrate. For several days, unbaited minnow traps were used to remove any stickleback from the constructed enclosures prior to experimental setup. Disturbance during enclosure setup, combined with the minnow traps, also ensured that fish predators were either absent or very rare in the enclosures (none were observed). However, aerial predation by piscivorous birds (terns or kingfishers) could contribute to performance effects.

Stickleback for the experiment were collected with unbaited minnow traps over several consecutive days from each of the three populations (Inlet, Lake, and Outlet). These fish were collected from a variety of locations near the experimental sites, but did not include the fish removed from the enclosures. We retained adult-size fish (min. ca. 50 mm total length) for the experiment – because they (as opposed to juveniles) are more likely to survive the tagging procedure and they could not escape from the enclosures. Adults are known to disperse in nature, including between lake and stream habitats (Bolnick et al. 2009; Moore and Hendry 2009), but the extent of juvenile dispersal is not known. Captured fish were transferred to 100L aquaria in the laboratory and held for a few days to conduct the pre-experimental procedures (see below) and to ensure their health prior to release into the enclosures.

Seventy-five fish from each population were haphazardly assigned to each of three experimental groups intended for the different enclosures. Prior to release into the enclosures, the fish were briefly anaesthetized with MS222, photographed with a digital camera (Nikon coolpix; Nikon Inc., Tokyo, Japan) on their left side, weighed on an electronic balance (to the nearest 0.01 g), and individually marked with coded wire tags (CWTs; North West Marinetechnology, Inc., Shaw Island, WA). The tags were inserted by injection into the muscle tissue on the left side of the body in front of the first dorsal spine. Although CWTs have not previously been used for stickleback, they are routinely used in a broad range of other fishes. Survival upon tagging was initially tested on stickleback in the laboratory, and no evidence was found for tagging-induced mortality. To ensure survival after handling, the fish were maintained overnight in holding tanks before being released to the enclosures.

Twenty-five fish of each of the three types were placed in each of the enclosures (i.e., 75 fish per enclosure, total *N* = 225) late May and left undisturbed for 21 days. This length of time has been used in several previous enclosure studies with stickleback (Hatfield and Schluter 1999; Hendry et al. 2002; Bolnick et al. 2010), where it has proven sufficient to reveal phenotype-specific performance differences. After this period, unbaited minnow traps were used intensively over several days to recapture the remaining fish (the tannic water in the Misty system prevented individual targeting for capture). The rarity of captures at the end of this period suggests that all or most of the surviving fish were recaptured. These fish were weighed (to nearest 0.01 g), euthanized with an overdose of MS222, photographed, and preserved in 95% ethanol. In the laboratory, they were dissected to determine sex and maturity status (male, female, and immature).

Of the released fish, 186 (82.7%) survived the experiment (i.e., were recaptured). Of these, 103 (55.4%) were males, 66 (35%) females, and 17 were immature (9.1%). The experimental period (late May) coincides with the breeding season of both Misty lake and stream stickleback and by the end of the experiment, 11 of 28 (39.2%) Inlet females (seven in the inlet, three in the lake, and two in the outlet enclosure), three of 12 (25%) Lake females (of which two in inlet and one in lake enclosure) were gravid. None of the 25 Outlet females was gravid. One Lake fish and one Outlet fish were clearly parasitized by Schistocephalus.

### Statistical analyses

Our two response variables (fitness or “performance” measures) were survival and mass change (individual mass of a survivor at the end of the experiment minus its mass at the beginning of the experiment). Log(mass change) was analyzed with analyses of variance (ANOVA) and analyses of covariance (ANCOVA) in Proc Mixed in SAS 9.3 (SAS Inc., Carry, NC) (details below). Fixed factors were sex (male, female, or immature), ecotype (Lake, Inlet, and Outlet), environment (lake, inlet, and outlet), and the ecotype × environment interaction. To avoid bias owing to gravid state or parasitism, analyses of log(mass change) were conducted excluding the gravid (*N *= 14) and parasitized (*N *= 2) individuals.

Survival was analyzed with generalized linear models with a logit link function and binomial error structure (Proc Genmod in SAS 9.3). Fixed factors were ecotype (Lake, Inlet, and Outlet), environment (lake, inlet, and outlet), and ecotype × environment interaction. Survival analyses did not include sex, because fish could not always be reliably sexed before transfer to enclosures.

Log(initial mass) was used as a covariate in analyses of both survival and mass change to assess and control for effects of initial size on performance. We first conducted a full model including all three ecotypes and environments. Because our specific hypotheses relate to the Lake versus Inlet and the Lake versus Outlet contrasts, separate analyses were conducted within each ecotype contrast: one analysis considered Lake and Inlet fish in lake and inlet enclosures, and the other considered Lake and Outlet fish in lake and outlet enclosures. (Analyses of the Outlet-Inlet contrast are provided in the Supporting Information).

In these analyses, a significant main effect of ecotype would indicate that ecotypes differ in overall performance (independent of the environment), a significant main effect of environment would indicate that environments differ in their effects on stickleback performance (independent of ecotype), and a significant ecotype × environment interaction would indicate that performance differences between the ecotypes depended on the specific testing environment. This last effect is the one most relevant for testing environment-dependent selection against migrants. To quantify the *magnitude* of selection against migrants, differences in survival between local and immigrant ecotypes in a given environment were calculated as (*W*_LOC_−*W*_MIG_)/*W*_SITE_, where *W*_LOC_ *and W*_MIG_ are survival of local and immigrant individuals and *W*_SITE_ is average survival across both ecotypes at a given site. These estimates are equivalent to those used by Hereford (2009) in his meta-analysis of local adaptation in reciprocal transplant experiments. We only calculated differences for survival as survival is more directly linked to fitness.

## Results

### Survival

**Figure 2:**
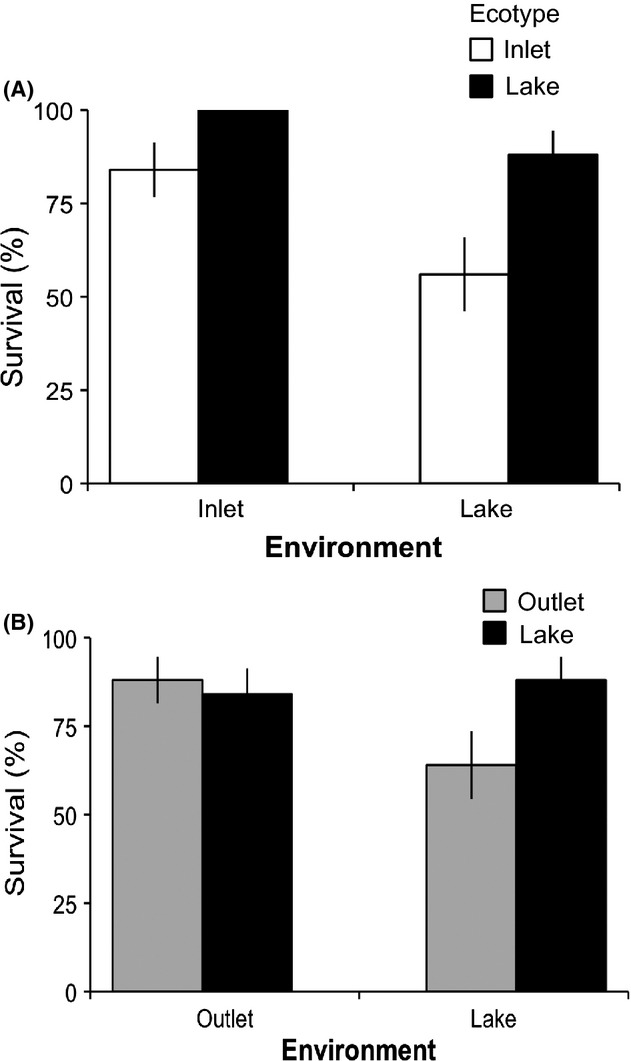
Survival mean ± SE of (A) Inlet and Lake stickleback in inlet and lake enclosures and (B) Outlet and Lake stickleback in outlet and lake enclosures.

In the full model, significant main effects revealed that survival differed among the environments and ecotypes (Table 1; Table S1). However, the lack of a significant ecotype × environment interaction meant an absence of support for reciprocal local adaptation. In the Lake-Inlet contrast, survival of both ecotypes was lower in the lake than in the inlet, and survival of Inlet fish was lower than survival of Lake fish in both environments, but also in this model there was no evidence for an ecotype × environment interaction (Table 1, Fig. 2A). In the Lake-Outlet contrast, survival of Lake and Outlet fish was similar in the outlet, whereas in the lake survival of Outlet fish was lower than that of the Lake fish (Fig. 2B). However, none of the main effects or the ecotype × environment interaction was significant (Table 1). In the full model, initial size was positively correlated with survival (log(*b)*: 8.61 ± 3.23), but the above results remained the same with or without size as a covariate (not shown). Initial size did not affect survival differences in the Lake-Inlet contrast (Table 1).

**Table 1:** Generalized linear models of survival for (A) three ecotypes (Inlet, Outlet, and Lake) of stickleback in three environments (inlet, outlet, and lake) with initial mass as covariate, (B) Lake versus Inlet (in lake and inlet environments), and (C) Lake versus Outlet (in lake and outlet environments).

Source	(A) All ecotypes			(B) Lake – Inlet		(C) Lake – Outlet	
	df	χ^2^	*P*	χ^2^	*P*	χ^2^	*P*
Ecotype	2	7.90	**0.019**	10.17	**0.001**	0.05	0.816
Environment	2	18.53	**<0.001**	8.05	**0.005**	2.34	0.127
Ecotype × Environment	4	5.55	0.236	1.26	0.261	2.15	0.142
Initial mass	1	7.41	**0.007**	0.21	0.649	7.81	**0.005**

Significant effects (*P* < 0.05) are highlighted in bold.

### Changes in mass

**Figure 3:**
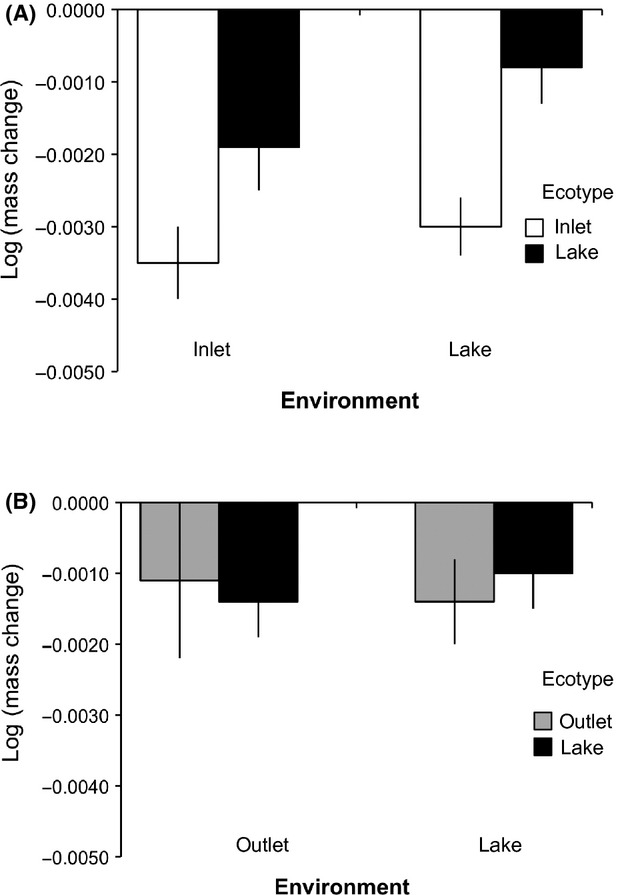
Log(mass change/day) of (A) Inlet and Lake stickleback in inlet and lake enclosure and (B) Outlet and Lake stickleback in outlet and lake enclosure. Values represent LSmeans ± SE from models with initial mass as a covariate.

Most fish lost some mass during the course of the experiment (Fig. 3), indicating that resources were limited – hence strengthening any potential resource competition-mediated effects. Overall, mass loss was influenced by the environment, the ecotype, and the ecotype × environment interaction (Table 2, Fig. S1). In the Lake-Inlet contrast, Inlet fish lost more mass than Lake fish in both environments, and the environments did not differ in their main effect on mass loss (Fig. 3A). The relatively greater mass loss of Inlet fish appeared to be greater in the lake (Fig. 3A), but the ecotype × environment interaction was not significant (Table 2). In the Lake-Outlet contrast, no significant main effects or interactions were detected (Table 2), and no hint of ecotype-dependent effects was seen in inspection of the data (Fig. 3B).

**Table 2:** Analyses of covariance on daily body mass change for (A) all three ecotypes (Inlet, Outlet, and Lake) of stickleback in three environments (inlet, outlet, and lake), (B) Lake versus Inlet fish in the lake versus inlet environments, and (C) Lake versus Outlet fish in the lake versus outlet environments. Denominator degrees of freedom = 154, 58 and 71, respectively.

Source	(A) All ecotypes			(B) Lake – Inlet		(C) Lake – Outlet	
	ndf	*F*	*P*	*F*	*P*	*F*	*P*
Ecotype	2	9.51	**<0.001**	17.16	**<0.001**	0.02	0.875
Environment	2	12.25	**<0.001**	2.99	0.089	0.01	0.915
Ecotype × Environment	4	2.60	**0.038**	0.48	0.489	0.67	0.416
Sex	2	1.78	0.172	0.45	0.642	1.62	0.206
Initial mass	1	31.47	**<0.001**	9.00	**0.004**	9.62	**0.003**

Significant effects (*P* < 0.05) are highlighted in bold.

### Selection against migrants

Inlet immigrants had lower survival than local (Lake) fish in the lake (0.44), suggesting the possibility of selection against migrants. In contrast, Lake immigrants had higher survival than local (Inlet) fish in the inlet (−0.17), suggesting no selection against migrants. In the Lake-Outlet contrast, there was no evidence for selection against Lake migrants in the outlet (0.05), but Outlet migrants had lower survival than local (Lake) fish in the lake (0.32).

## Discussion

Selection against migrants can be a powerful reproductive barrier (Hendry 2004; Nosil et al. 2005). We tested the strength of this barrier in the Misty lake watershed by comparing strongly divergent Lake and Inlet stickleback and weakly divergent Lake and Outlet stickleback. We first predicted that, mirroring the patterns of trait divergence, Lake and Inlet stickleback should differ in their performance (i.e., in the fitness metrics survival and mass change), whereas Lake and Outlet stickleback should not. We then predicted that, given local adaptation, Lake fish should perform better than Inlet fish in the lake and Inlet fish should perform better than Lake fish in the inlet (selection against migrants), whereas Lake and Outlet fish should perform similarly in both the lake and outlet environments.

### Selection against migrants?

Consistent with our first prediction, Lake and Inlet fish showed differences in survival and mass change, whereas Lake and Outlet fish did not. These findings confirm that the numerous traits known to differ between the Lake and Inlet fish – but not between the Lake and Outlet fish (see Introduction) – do indeed influence fitness components. As expected then, trait divergence causes fitness divergence, and a lack of trait divergence constrains fitness divergence. The latter result increases support for previous inferences that gene flow constrains adaptive divergence in the Lake-Outlet pair (Hendry et al. 2002; Moore et al. 2007; Sharpe et al. 2008; Berner et al. 2008, 2009; Roesti et al. 2012). Given this fit with previous expectations for the Lake-Outlet comparison, we turn our attention to the conundrum presented by the Lake-Inlet comparison.

The clearest outcome for selection against migrants would be a situation where each ecotype performs better in its native environment than does the other ecotype (Schluter 2000). This pattern we clearly did not find, suggesting that – at face value – local adaptation is weak or absent (sensu Kawecki and Ebert 2004) and that selection against migrants would not contribute to reproductive isolation. Closer examination of the data, however, yields a more nuanced interpretation. First, Inlet fish indeed performed worse in the lake than did Lake fish: viability selection against migrants was estimated to be 0.44, a value higher than the average viability-based estimates of local adaptation (ca. 0.25) in Hereford's (2009) meta-analysis. However, Inlet fish also performed worse in the inlet than Lake fish did (−0.17), which suggests that Inlet fish simply do poorly everywhere (see more below). The Lake-Inlet differences in survival and mass change appear to be somewhat higher in the lake than the inlet –indicating asymmetric (Inlet to Lake) selection against migrants.

In the other direction (Lake to Inlet), we have no evidence for selection against migrants, which would seem to contradict expectations that adaptation to one environment (here the lake) should decrease relative fitness in another environment (here the Inlet). Perhaps that expectation is too simple, however, given that a similar situations (one type performs better than the other type in both environments and where *trait* divergence is not correlated with degree of local adaptation), appears relatively frequent across different study systems (reviewed in Hereford 2009). Moreover, in a number of instances experimental adaptation to one environment has not lead to a trade-off in performance in other environments (e.g., Bennett and Lenski 2004; reviewed in Hereford 2009).

Taken together, these observations suggest that more studies should explicitly consider *asymmetric* selection against migrants. Of course, it remains possible that we would have found selection against Lake fish in the Inlet had we looked at a different age class (e.g., juveniles), a different season (e.g., winter), or a different fitness surrogate (e.g., reproductive success or fecundity). With regard to juvenile performance, further studies are clearly warranted as nothing is currently known of either juvenile dispersal or selection on juveniles. With regard to seasonal effects, our experiment coincided with the breeding season of both Lake and Inlet fish (A. P. Hendry, unpubl. data), but a somewhat larger proportion of Inlet (than Lake) females was gravid at the end of the experimental period. It is therefore possible that the overall greater mass loss of Inlet fish across environments reflects their investment in reproductive activities. Fecundity-mediated divergent selection could certainly also contribute to selection against migrants, given differences in maternal greater investment in this system (Baker et al. 2013). Finally, we might have detected symmetric selection against migrants had we conducted the study over a longer time (e.g., over multiple years) and using a different experimental setup (e.g., multiple replicated enclosures). However, the duration, timing, and fitness metrics in our study were quite similar to previous stickleback studies (of the benthic/limnetic pairs) that have documented symmetric selection against migrants (e.g., Hatfield and Schluter 1999; Rundle 2002), and the large size of enclosures with many interacting individuals used here mimics a natural migration event. For this reason, we are confident in concluding that viability selection against migrants is certainly less strong and symmetrical in the lake/stream system than it is in the benthic/limnetic systems.

### Mosaic reproductive isolation

In the case of an asymmetric reproductive barrier, symmetric overall reproductive isolation might be achieved through additional reproductive barriers. For example, in two species of *Ischnura* damselflies, reproductive isolation in one direction is prevented primarily by strong premating isolation, whereas reproductive isolation in the other direction results from a combination of multiple reproductive barriers (Sánchez-Guillén et al. 2012). Another example comes from Pacific Ocean and Japan Sea stickleback, where crosses in one direction are rare due to female mate choice, and crosses in the other direction lead to hybrid male sterility (Kitano et al. 2009). In the lake/stream situation, a likely reproductive barrier acting in the opposite direction (Lake to Inlet) to asymmetric selection against migrants (Inlet to Lake) is the response to water current. For instance, studies in a different watershed have shown that Lake stickleback rarely ventured into an inlet stream (Bolnick et al. 2009). Pointing toward a similar effect in the Misty system, Lake but not Inlet fish are displaced downstream when released into the inlet (Hendry et al. 2002). Thus, selection against migrants from the Inlet to the Lake might be complemented by a lack of migration from the lake to the inlet. The first of these effects is likely enhanced by the small size of the Inlet population in relation to the Lake population (Hendry et al. 2002), and the second effect is likely enhanced by the narrow contact zone between the Lake and the Inlet populations.

In summary, the combination of selection against migrants in one direction and the lack of migrants in the other direction might generate the observed very low gene flow and thus resolve “the conundrum of missing reproductive isolation” (Räsänen et al. 2012). In addition, the resulting rarity with which individuals would successfully migrate between these environments suggests that Lake and Inlet fish would not experience much selection to mate with their own type (i.e., reinforcement), which would then explain the lack of assortative mating observed in laboratory trials (Raeymaekers et al. 2010; Räsänen et al. 2012). Alternatively, the lack of assortative mating found in laboratory experiments could result from the use of artificially raised common garden fish – whereas sexual imprinting (Kozak et al. 2011) or other aspects of phenotypic plasticity might be important.

In addition to selection against migrants and limited migration, a number of other barriers might contribute to reproductive isolation in the Misty system and in other lake/stream pairs. As just one example, reproductive timing differs between lake and stream fish in at least some systems (J. S. Moore, D. Hanson, & A. P. Hendry, unpubl. data). In addition, hybrids often have intermediate phenotypes for adaptive traits (Raeymaekers et al. 2010; Berner et al. 2011) that might be poorly suited for one or both parental environments – although this has yet to be formally tested. In contrast, genetic incompatibilities seem weak or absent, given that lake/stream fish can successfully be crossed and produce viable hybrids (Lavin and McPhail 1993; Berner 2011). Overall, then, work on lake/stream stickleback suggests that a mosaic of multiple asymmetric reproductive barriers might be the reason why gene flow is often limited even when the populations are apparently not isolated in some single reproductive barrier.

### Implications

Threespine stickleback are one of the canonical examples of ecological speciation – a reputation largely built on studies of sympatric benthic–limnetic pairs and anadromous–freshwater pairs (reviews: McKinnon and Rundle 2002; Boughman 2006; Hendry et al. 2009). These studies helped to build general expectations that ecological speciation is very common and powerful (Schluter 2000; Rundle and Nosil 2005; Nosil 2012). As more studies of stickleback have been conducted, however, ecological speciation now seems far from inevitable: (1) sympatric benthic–limnetic divergence is not present outside of six small lakes (Bolnick 2011) and is prone to collapse (Taylor et al. 2006), (2) a number of other population pairs do not show strong assortative mating (Jones et al. 2008; Raeymaekers et al. 2010; Räsänen et al. 2012), and (3) parapatric divergence (such as between lakes and streams) ranges from very high to very low (Berner et al. 2009, 2010; Roesti et al. 2012).

These new and more variable results indicate that ecological speciation might be strongly constrained in many instances, that the reproductive barriers might vary dramatically in type and strength across replicate systems, and that a mosaic of barriers might be necessary for substantial progress toward ecological speciation. We suspect that similar results attend many other natural systems, such as *Timema* walking sticks (Nosil 2007), European whitefish (e.g., Siwertsson et al. 2010), Arctic charr (e.g., Jonsson and Jonsson 2001; Wilson et al. 2004), and *Heliconious* butterflies (Merrill et al. 2011). A thorough understanding of this mosaic nature of reproductive isolation will be possible only through the study of many different populations/systems and the examination of multiple reproductive barriers.

## Supporting Information

**Table S1.** Analyses of survival and mass change for Inlet–Outlet contrast. **Figure S1.** Survival and mass change of all three ecotypes in all three environments.
