# Supplementary material for: Asymmetric reproductive barriers and mosaic reproductive isolation: insights from Misty lake–stream stickleback
Source: Ecol Evol. 2014 Mar 10;4(7):1166–75. doi: 10.1002/ece3.1012 (PMC3997330; doi:10.1002/ece3.1012)
Supplement: Supplementary file 1 — Table S1. Analyses of survival and mass change for Inlet–Outlet contrast. Figure S1. Survival and mass change of all three ecotypes in all three environments. [file ece30004-1166-sd1.docx]

**Supplementary Information**

**Table S1.** A) Generalized linear model of survival and B) Analysis of covariance of mass change of Inlet and Outlet threespine stickleback in inlet and outlet enclosures. Ddf for mass change is = 71.

**A) Survival B) Mass change**

*df χ^2^ P F P*

Ecotype 1 2.46 0.117 1.91 0.171

Environment 1 0.83 0.364 29.18 **<0.001**

Ecotype × Environment 1 0.47 0.492 2.73 0.103

Sex 2 - - 1.08 0.344

Initial weight 1 1.55 0.214 22.12 **<0.001**

**FigS1.** Räsänen and Hendry.


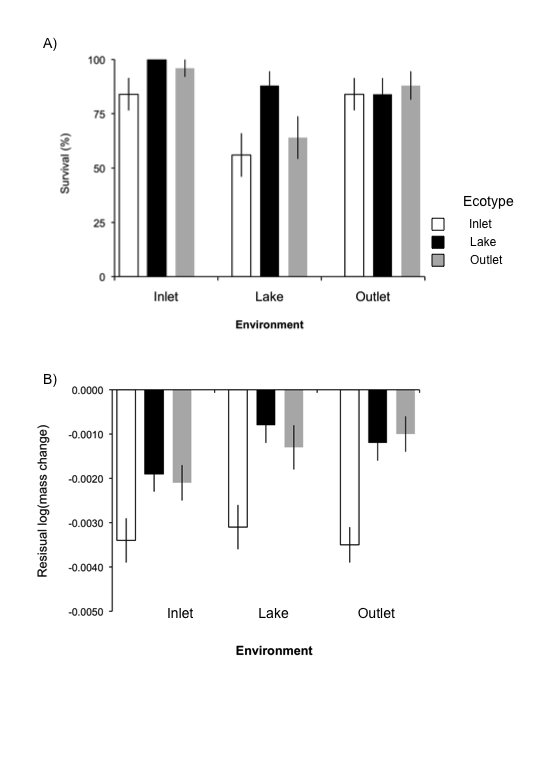


**Figure S1.** A) Survival mean ± S.E. and b) LS means ± S.E. of Log(mass change/day) of three threespine stickleback ecotypes (Inlet, Outlet, Lake) in experimental enclosures the three environments (Inlet, Outlet, Lake). In B values are LS means from models where initial mass was included as a covariate.
